# Validation of rK39 immunochromatographic test and direct agglutination test for the diagnosis of Mediterranean visceral leishmaniasis in Spain

**DOI:** 10.1371/journal.pntd.0006277

**Published:** 2018-03-01

**Authors:** Mathieu Bangert, María D. Flores-Chávez, Ivonne P. Llanes-Acevedo, Carolina Arcones, Carmen Chicharro, Emilia García, Sheila Ortega, Javier Nieto, Israel Cruz

**Affiliations:** 1 European Public Health Microbiology Training Program (EUPHEM), European Centre for Disease Control and Prevention, Stockholm, Sweden; 2 WHO Collaborating Centre for Leishmaniasis, National Centre for Microbiology, Instituto de Salud Carlos III, Majadahonda, Spain; Istituto Superiore di Sanità, ITALY

## Abstract

**Background:**

Visceral leishmaniasis (VL), the most severe form of leishmaniasis, is endemic in Europe with Mediterranean countries reporting endemic status alongside a worrying northward spread. Serological diagnosis, including immunochromatographic test based on the recombinant antigen rK39 (rK39-ICT) and a direct agglutination test (DAT) based on the whole parasite antigen, have been validated in regions with high VL burden, such as eastern Africa and the Indian subcontinent. To date, no studies using a large set of patients have performed an assessment of both methods within Europe.

**Methodology/Principal findings:**

We selected a range of clinical serum samples from patients with confirmed VL (including HIV co-infection), Chagas disease, malaria, other parasitic infections and negative samples (n = 743; years 2009–2015) to test the performance of rK39-ICT rapid test (Kalazar Detect Rapid Test; InBios International, Inc., USA) and DAT (ITM-DAT/VLG; Institute of Tropical Medicine Antwerp, Belgium). An in-house immunofluorescence antibody test (IFAT), was included for comparison. Estimated sensitivities for rK39-ICT and DAT in HIV-negative VL patients were 83.1% [75.1–91.2] and 84.2% [76.3–92.1], respectively. Sensitivity was reduced to 67.3% [52.7–82.0] for rK39 and increased to 91.3% [82.1–100.0] for DAT in HIV/VL co-infected patients. The in-house IFAT was more sensitive in HIV-negative VL patients, 84.2% [76.3–92.1] than in HIV/VL patients, 79.4% [73.3–96.2]. DAT gave 32 false positives in sera from HIV-negative VL suspects, compared to 0 and 2 for rK39 and IFAT, respectively, but correctly detected more HIV/VL patients (42/46) than rK39 (31/46) and IFAT (39/46).

**Conclusions/Significance:**

Though rK39-ICT and DAT exhibited acceptable sensitivity and specificity a combination with other tests is required for highly sensitive diagnosis of VL cases in Spain. Important variation in the performance of the tests were seen in patients co-infected with HIV or with other parasitic infections. This study can help inform the choice of serological test to be used when screening or diagnosing VL in a European Mediterranean setting.

## Author summary

Visceral leishmaniasis is the most severe form of leishmaniasis, a disease transmitted through the bite of an infected sandfly. Although the biggest burden of leishmaniasis is in eastern Africa and the Indian subcontinent, the disease is also endemic in parts of Europe. Previous studies have looked at performance of diagnostic methods, but not in great detail on samples derived from a European setting. Using a large set of samples from a national reference laboratory in Madrid, Spain, we assessed a leishmaniasis rapid test and a direct agglutination test for serological diagnosis of visceral leishmaniasis in Europe. Both tests were effective at diagnosing VL, but important differences were seen when testing patients co-infected with HIV or with other parasitic infections. This study can help inform which diagnostic tests are suitable for use in a European Mediterranean setting.

## Introduction

Visceral leishmaniasis (VL) is a life-threatening disease caused by protozoan parasites of the *Leishmania donovani* complex. It is widely endemic in South America, eastern Africa and Asia as well as in the Mediterranean basin [1]. More than 500 million people are at risk of acquiring leishmaniasis worldwide, with approximately 90% of the cases arising in rural areas of Bangladesh, Brazil, Ethiopia, India, Somalia, South Sudan and Sudan [2]. In Europe, nine countries report cases of VL annually accounting for less than 2% of the global burden [3], where cases are mostly confined to the Mediterranean countries, but a spread towards northern Europe is being reported as a result of a range of factors, including vector and parasite migration, and changes to the environment and climate [4]. In Spain, a VL outbreak of unprecedented magnitude occurred in the southwest of the capital Madrid between 2009–2013 [5,6], and the country was recently listed among the top 14 VL high-burden country [2].

Facing a possible (re-)emergence of leishmaniasis in Europe, it is important for national public health institutions to have established guidelines for clinical diagnosis of VL to support primary health care and epidemiological surveillance [3,7]. Parasitological confirmation through culturing and/or microscopy remains the gold standard for diagnosis, and gives the clearest indication of parasitic infection. The sensitivity of parasitological confirmation, however, depends on the sample used, where spleen and bone marrow aspirates yield the best results but these are obtained through invasive sampling procedures, with inherent complications, besides presenting variable sensitivity [8]. In addition, the absence of parasites in tissue sample does not necessarily indicate absence of infection. Nucleic acid amplification tools have shown to be more sensitive than microscopy or culture for VL diagnosis, even when using peripheral blood samples [9]. This technology is already available in many hospitals and reference centers in VL-endemic countries in Europe; unfortunately there is a consistent lack of standardization and a very high number of different protocols [9].

Serological tools provide a good diagnostic accuracy as long as they are used in combination with a standardized clinical case definition for VL [1]. Serological tests vary in the target antigen (whole parasite or recombinant protein), ease-of-use (rapid dipstick or necessity for some laboratory infrastructure), sensitivity, specificity, and cost. Underlying HIV infections, or other forms of immunosuppression, however, can affect their sensitivity [10].

The rK39 immunochromatographic test (rK39-ICT) and the direct agglutination test (DAT) have been widely validated in the VL endemic regions of eastern Africa and the Indian subcontinent, with rK39-ICT demonstrating varying sensitivity and specificity depending on the geographical setting [11–13].To our knowledge, no studies using a large set of patients have performed an assessment of both methods on human samples within Europe. To establish evidence on serological VL diagnostic performance in this region, we assessed the sensitivity and specificity of rK39-ICT, DAT and IFAT using historical serum samples collected in Spain from 2009–2015.

## Methods

### Ethics statement

The serum samples used in this study are anonymized and are part of a registered collection, as described below. No ethical approval was required.

### Study site

The study was conducted at the WHO Collaborating Centre for Leishmaniasis, National Centre for Microbiology, Instituto de Salud Carlos III, Madrid, Spain (WHOCCL-ISCIII), which is also the national reference laboratory for leishmaniasis.

### Characteristics of sera and study design

We used historical serum samples stored at -70°C at the WHOCCL-ISCIII. These samples are part of a collection registered at the National Biobank Register-Section Collections, Spain, with collection Reference ID: C.0000898. The serum samples in the collection are anonymized.

Samples from suspected VL cases are derived from patients with clinical suspicion of VL as defined in the protocol of the Spanish national network for epidemiological surveillance [14], and were referred from different hospitals to the WHOCCL-ISCIII for diagnosis from 2009–2015. Briefly, a suspected VL case in Spain is defined as a patient who presents with irregular prolonged fever plus splenomegaly and/or weight loss, which may be accompanied by hepatomegaly, lymphadenopathy, leukopenia, anemia and thrombocytopenia. Each suspected cases had multiple samples (whole blood, serum, bone marrow) taken to facilitate diagnosis. While in this study only serum samples were tested, we used all laboratory and clinical results available from each patient to classify them as “case” or “non-case”, and therefore define the reference diagnostic result (see *parasitological confirmation* below). Samples from VL suspects were further divided according to the HIV status of the patients. In addition, we chose samples from patients who were diagnosed with malaria, Chagas disease or other parasitic infections, as well as serum from healthy individuals (blood donors) from Spain, Belgium and Germany. All samples were anonymized and diagnostic test operators were blinded to the nature of the serum sample.

### Serological diagnostic tests

The rK39-ICT (Kalazar Detect Rapid Test, Inbios International Inc., WA, USA), and the DAT with freeze-dried antigen (ITM-DAT/VL; Institute of Tropical Medicine, Antwerp, Belgium) were performed according to manufacturer’s instruction; with 20 and 1 μl serum respectively. DAT was performed by using the screening method, samples with a titer ≥ 1:3200 were considered positive [15]. An *in house* IFAT was performed by following a standard method [16]: the antigen was prepared from promastigotes of the *L*. *infantum* international reference strain MHOM/FR/78/LEM-75. Antibody binding was detected using fluorescein isothiocyanate-conjugated sheep anti-human immunoglobulin G (heavy and light chains). One μl serum was used. The threshold titer for positivity was ≥1/80. Test results were interpreted and recorded on a standardized form by at least two observers at the minimum reading times, where each observer was blinded to the other’s reading. Any test returning an invalid result or lack of agreement between observers was repeated.

### Parasitological confirmation

As part of routine diagnosis VL suspect patients are tested at the WHOCCL-ISCIII by nested PCR of blood and bone marrow samples, bone marrow Giemsa microscopy and blood and bone marrow NNN culture, following procedures described elsewhere [17,18]. A serum sample from a VL suspect was defined as pertaining to a case when there was parasitological confirmation of *Leishmania* in blood and/or bone marrow aspirate in samples taken within 21 days before or after the serum sample was taken.

### Statistical analysis

The statistical software R was used with the ‘epiR’ package to determine sensitivity, specificity, positive and negative predictive values [19,20]. Exact binomial confidence limits were calculated for test sensitivity, specificity, and positive and negative predictive values.

STARD checklist and workflow are provided as supplementary materials, S1 Table and S1 Fig respectively.

## Results

**Fig 1:**
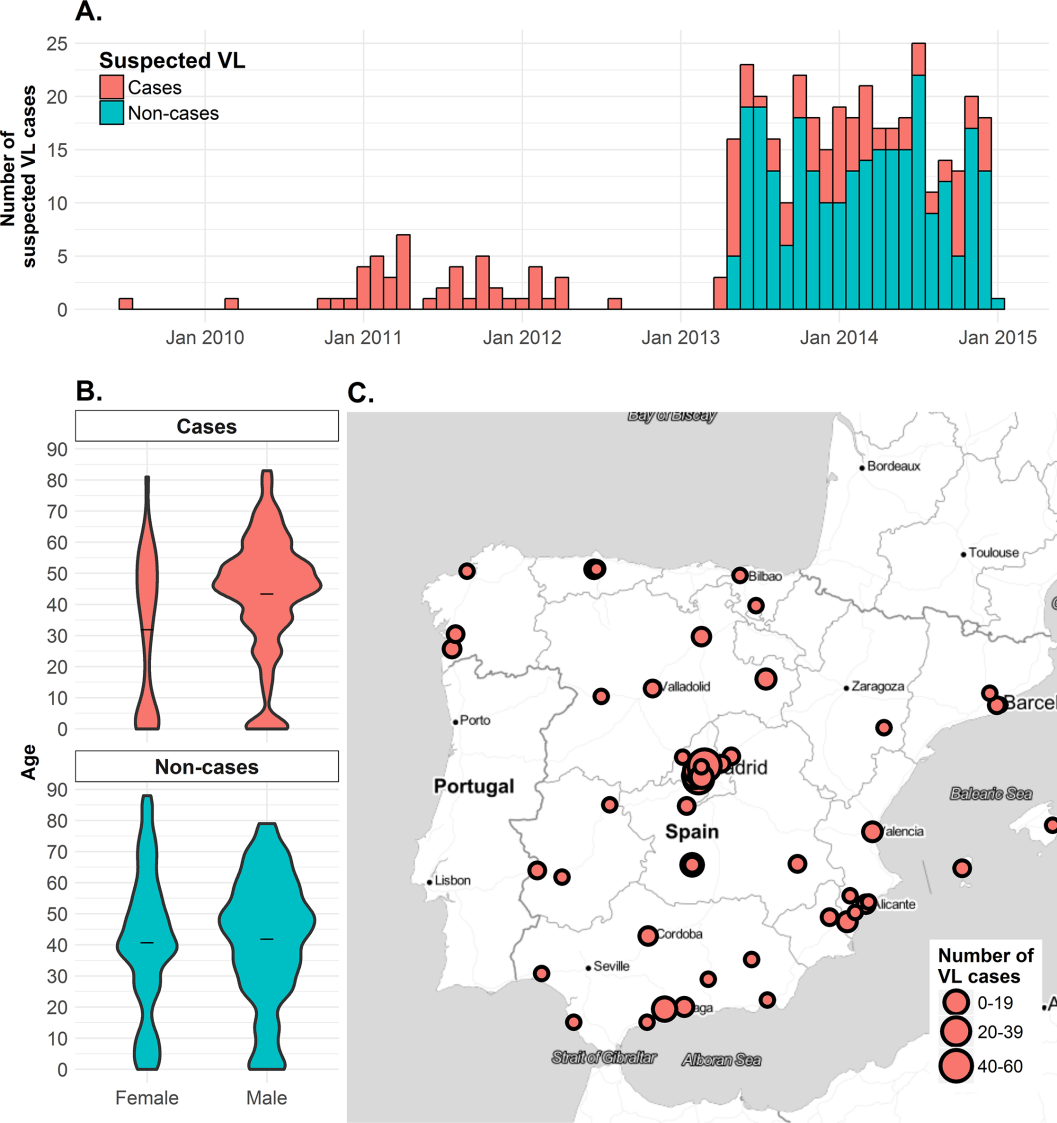
Timeline and demographics of suspected visceral leishmaniasis (VL) cases tested at the WHOCCL-ISCIII, Spain, 2009–2015. Panel A shows the number of VL cases and non-cases tested by month. Panel B shows the age and sex distribution of suspected VL cases and non-cases. Panel C maps the location of health centres in Spain where VL cases sought diagnosis (circles) and the number of VL cases per health centre (size of circle). Map tiles by Stamen Design, under CC BY 3.0. Data by OpenStreetMap, under ODbL.

A total of 743 samples from 2009–2015 were tested, of which 405 were suspected VL cases, and 338 samples as control group. Most samples were taken from March 2013 to January 2015 (Fig 1A) in hospitals from different regions in Spain, mostly from Madrid and the Mediterranean coast (Fig 1C).

Seventy percent of suspected cases were male, with an average age of 41 years (Fig 1B). Seventy-six patients were HIV positive, while 12 patients had immunosuppression related to organ transplantation (n = 11) and Crohn’s disease (n = 1). The composition of serum samples and the diagnostic test results is detailed in Table 1.

**Table 1:** Type and number of samples used in this study and diagnostic outcome from each test assessed.

		rK39-ICT		DAT		IFAT		Parasitological confirmation*	
	N	pos	neg	pos	neg	pos	neg	pos	neg
Suspected VL	405	110	295	246	159	114	291	0	264
Cases	141	110	31	122	19	112	29	141	0
Non-cases	264	0	264	37	227	2	262	0	264
Control groups	338	1	337	4	334	15	323	NA	NA
Chagas disease	51	0	51	0	51	15	36	NA	NA
Malaria	55	1	54	2	53	0	55	NA	NA
Other parasitoses	57	0	57	2	55	0	57	NA	NA
Spanish blood donors	142	0	142	0	142	0	142	NA	NA
German blood donors	13	0	13	0	13	0	13	NA	NA
Belgian blood donors	20	0	20	0	20	0	20	NA	NA
Total	743	111	632	163	580	129	614		

*Positive parasitological confirmation by either nested PCR in blood or bone marrow, Giemsa microscopy in bone marrow aspirate or NNN-culture of blood and/or bone marrow samples.; NA = not applicable, as parasitological confirmation for *Leishmania* infection was not performed in this group; pos = positive results; neg = negative result.

The sensitivity, specificity, positive and negative predictive values of each test are given in Table 2.

**Table 2:** Performance of rK39, DAT and IFAT on suspected visceral leishmaniasis samples in Spain, 2009–2015. Sensitivity (Sn), specificity (Sp), positive predictive value (PPV), negative predictive value (NPV) with 95% confidence intervals [].

	rK39-ICT		DAT		IFAT	
**Sn**	78.0%	[70.8–85.2]	86.5%	[80.5–92.5]	79.4%	[72.4–86.4]
**Sp**	100.0%	[99.8–100.0]	85.9%	[81.6–90.3]	99.2%	[98.0–100.0]
**PPV**	100.0%	[99.5–100.0]	76.7%	[69.8–83.6]	98.2%	[95.4–100.0]
**NPV**	89.5%	[85.8–93.1]	92.3%	[88.7–95.8]	90.0%	[86.4–93.6]

Sn: Sensitivity; Sp: specificity; PPV: positive predictive value; NPV: negative predictive value.

A sub group analysis according to the HIV status of the VL suspect patients is shown in Table 3.

**Table 3:** Sub group analysis on HIV status. Performance of rK39, DAT and IFAT on suspected visceral leishmaniasis samples in Spain, 2010–2014. Sensitivity (Sn), specificity (Sp), positive predictive value (PPV), negative predictive value (NPV) with 95% confidence intervals [].

**HIV POSITIVE (n = 76)**	**rK39-ICT**		**DAT**		**IFAT**	
** Sn**	67.3%	[52.7–82.0]	91.3%	[82.1–100]	79.4%	[73.3–96.2]
** Sp**	100.0%	[98.3–100]	83.3%	[68.3–98.3]	99.2%	[98.3–100]
** PPV**	100.0%	[98.4–100]	89.3%	[79.5–99.2]	98.2%	[98.7–100]
** NPV**	66.7%	[51.8–81.5]	86.2%	[71.9–100]	90.0%	[67.1–95.0]
**HIV NEGATIVE** **(n = 329)**	**rK39-ICT**		**DAT**		**IFAT**	
** Sn**	83.1%	[75.1–91.2]	84.2%	[76.3–92.1]	84.2%	[76.3–92.1]
** Sp**	100.0%	[99.8–100]	86.3%	[81.7–90.9]	86.3%	[81.7–90.9]
** PPV**	100.0%	[99.4–100]	71.4%	[62.6–80.2]	71.4%	[62.6–80.2]
** NPV**	93.6%	[90.4–96.8]	93.1%	[89.5–96.7]	93.1%	[89.5–96.7]

Sn: Sensitivity; Sp: specificity; PPV: positive predictive value; NPV: negative predictive value.

### rK39-ICT

The estimated sensitivity of rK39 was 78.0% [70.8–85.2] for all 405 suspected VL patients. Of the 95 HIV-negative VL cases, 79 were correctly diagnosed by rK39 giving a sensitivity of 83.1% [75.1–91.2]. The sensitivity dropped to 67.3 [52.7–82.0] in individuals with underlying HIV infection. Of the 602 negative samples (338 control subjects and 264 non-confirmed VL suspects), rK39 gave 1 false positive in a malaria patient.

### DAT

The estimated sensitivity of DAT was 86.5% [80.5–92.5] for all 405 suspected VL patients. The sensitivity of DAT in HIV-negative VL suspects was 84.2% [76.3–92.1], rising to 91.3% [82.1–100.0] in individuals with underlying HIV infection. Of the 602 negative samples (338 control subjects and 264 non-confirmed VL suspects), DAT gave false positive results for 41 serum samples, including 2 individuals with malaria and two individuals with other parasitic infections.

### IFAT

The estimated sensitivity of IFAT was 79.4% [72.4–86.4] for all 405 suspected VL patients. The sensitivity of IFAT in HIV-negative VL suspects was 84.2% [76.3–92.1] dropping to 79.4% [73.3–96.2] in VL suspects with HIV. Of the 602 negative samples (338 control subjects and 264 non-confirmed VL suspects), IFAT gave false positives for 2 non-confirmed VL suspects and 15 patients with Chagas disease.

## Discussion

In this study, we assessed the sensitivity and specificity of rK39-ICT, DAT and IFAT on a varied set of historical serum samples collected in Spain from 2009–2015. The diagnostic performance of rK39-ICT and DAT has been largely evaluated in highly endemic country settings [12], with variable results in different geographic locations for rK39-ICT [21], and the performance in a European setting remains largely unknown. A multicenter study compared different diagnostic tests using samples from 26 HIV-negative and 11 HIV-positive VL patients from southern France [22]. This study evaluated a different rK39-ICT (IT-LEISH Bio Rad Laboratories, France) and DAT (the same as in our study), among other serological tests, and obtained a sensitivity of 88.5% for both in HIV-negative VL patients and 54.5% for DAT and 81.8% for rK39-ICT in HIV/VL patients. In a study from Italy with a sample size of 94 patients with suspected VL (21 patients were confirmed VL cases), the reported sensitivity of rK39-ICT was 52.4%, using a different manufacturer than in our study [23]. These results differed from our study, which are more in agreement with other large scale evaluations of rK39-ICT and DAT that show higher sensitivity for DAT in a setting with high HIV-co-infection rate [24].

The evaluation of diagnostic tools for visceral leishmaniasis in Europe is important as the burden of VL remains an issue for European public health officials [3]. We began addressing this issue using a large assembly of samples. Our results show lower sensitivity estimates when compared to published results on serological assay evaluation in South East Asia, the Americas and eastern Africa regions. In a meta-analysis of diagnostic performance, the combined sensitivity estimates of DAT and the rK39-ICT were 94.8% and 93.9%, respectively [25], while in our study, the estimated DAT and rK39-ICT sensitivity was at 86.5% and 78.0%, respectively. In a WHO led evaluation of rK39-ICT, the sensitivity estimates varied greatly from region to region: 67.6% in eastern Africa, 84.7% in Brazil and 99.6% on the Indian subcontinent [21].

The different performance of serological tests between European samples and those tested elsewhere is most likely due to the epidemiological landscape. Patients residing outside of Europe will have different anti-VL immunoglobulin titers, different age patterns of infection, immune and/or nutritional background and/or are exposed to higher parasite diversity [21]. A study analyzing *L*. *donovani* strains from African and Asian origin revealed extensive genetic diversity in coding sequences of rK39 homologues, which may provide an explanation for the different performance of rK39-ICT across regions [26]. In the Mediterranean region VL is caused by different genetic variants of *L*. *infantum* [27,28], whether this has an effect on the performance of rK39-ICT would be an interesting subject of study.

Our study sample reflects a population living in a southern European member state where samples are routinely submitted for laboratory diagnosis after clinical suspicion of VL. Of the 405 samples submitted between 2009 and 2015 and analyzed in this study, 34% were classified as cases. To expand our sample population and assess the performance of serological tests with respect to cross-reactivity, we further selected confirmed VL negative serum samples with varying immunological exposures, including Chagas disease, caused by another member of the Trypansomatidae family, as well as German and Belgian blood donors who are less likely to have had previous parasite exposure.

Although sensitivity and specificity varied between diagnostic tests, we found that the in house IFAT, rK39-ICT (Kalazar Detect from InBios International, Inc.) and DAT (ITM-DAT/VL, Institute of Tropical Medicine, Antwerp) are valid for VL diagnosis in Europe. The choice of test, however, is according to the epidemiological context and intended application. In the context of VL in Europe we find three main applications for serological tests: seroprevalence studies, clinical diagnosis and outbreak response tools.

Seroprevalence studies involve large-scale screening of samples to determine the burden of disease in a given population. Previous prevalence studies in Europe on blood donors have used different immunological and/or molecular tests [29–31]. Based on our results, we find DAT performed best for seroprevalence studies, with the ability to batch process samples, the acceptable costs, and the specificity and sensitivity values at 86% and 85%, respectively.

For clinical diagnosis, the choice of test depends partly on the immunological status of the patient. In our study, co-infection with HIV reduced the sensitivity of rK39-ICT, making it less applicable for a point-of-care test in HIV individuals in Spain. During the community outbreak of VL in Madrid, 16 out of 160 reported VL cases (10%) had HIV [5], and could therefore have been missed if rK39 was used as sole diagnostic. In a series of 73 VL patients (66% immunocompetent) from that outbreak, another rK39-ICT (SD BIOLINE *Leishmania* Ab, Standard Diagnostics, Inc., South Korea) showed 67% sensitivity and 100% positive predictive value [32]. To our surprise, in our study the DAT showed higher sensitivity in HIV-positive patients. Although a higher sensitivity in this group is somehow unexpected, it is important to highlight that DAT has returned acceptable sensitivity in the diagnosis of VL in HIV-positive patients, being superior to other serological tests [10,24,33–35]. It is difficult for us to find an explanation to this, and it could be suggested that the observed discrepancy may be due to the difference in the number of patients in each group; being only relevant for rK39-ICT (the only test using a single antigen), for which the different performance according to the HIV status is especially marked. A study specifically designed to assess differences in the diagnostic performance of the tests according to the HIV status would be necessary to address this properly. We did not conduct a separate analysis in patients with other immunosuppressive conditions as previous studies have shown that the diagnostic sensitivity of serological tests is not decreased in patients receiving solid organ transplants, which were 11 out of 12 of our suspected VL cases with immunosuppressive conditions other than HIV [36–38].

During outbreak settings such as those seen in Madrid in 2009–2013, point-of-care tests like rK39-RDT have the benefit of portability, simplicity and the speed of result, allowing quick identification and control of infection clusters. In our study we used all laboratory (PCR, culture, serology) and clinical results available from each patient to classify them as “case” or “non-case”. The reported sensitivities of the serological tests included in this study justify the algorithm proposed for VL diagnosis in the WHO European region, where rK39-ICT is first used in VL suspected cases and can be complemented with other serological or parasitological tests to ensure accurate diagnosis [3]. The rK39-ICT is a simple, fast, commercially available test that uses a less invasive sample. The application of this test for VL diagnosis and subsequent treatment of confirmed cases with liposomal amphotericin B, the reference treatment for VL in the WHO European Region [3], has shown to be cost-effective for Mediterranean VL management in Morocco [39].

In terms of cross reactivity, we found that all true negative serum samples from blood donors from Belgium, Germany and Spain were diagnosed as negative for VL by DAT and rK39-ICT. Some false positive results were obtained with Chagas disease patients and those with other parasitic infections, this was particularly pronounced for the IFAT, a widely used serological test for VL diagnosis in Europe. This can be explained by serological cross reactivity between trypanosomatids [40]. In order to account for this, other infections such as Chagas disease, malaria or other parasites should be routinely discarded to increase diagnostic accuracy. This is particularly important in diagnosing a patient who has resided in or visited a country endemic for other parasitic disease, such as is common in the Spanish migrant populations [41].

To the best of our knowledge this is the first large-scale evaluation of rK39-ICT and DAT for VL diagnosis in Europe. These results can inform public health practitioners in the region on the strengths and limitations of serological diagnosis. In addition to serology, however, PCR diagnosis should always be considered for confirmation of infection, and for the added benefit that molecular characterization brings.

Finding appropriate diagnostic solutions to VL is not only important to contain the burden of this Neglected Tropical Disease, but it will also help in the implementation of the United Nations Sustainable Development Goal of Universal Health Coverage [42].

## Supporting information

S1 Table STARD checklist. Detailed list of items related to reporting of diagnostic accuracy studies.
(DOCX)

S1 Fig STARD workflow. Details on the classification and workflow for testing samples in this study.
(PPTX)

## References

[1] WHO Expert Committee on the Control of the Leishmaniases, World Health Organization, editors. Control of the leishmaniases: report of a meeting of the WHO Expert Committee on the Control of Leishmaniases, Geneva, 22–26 March 2010. Geneva: World Health Organization; 2010.

[2] World Health Organization. Leishmaniasis in high-burden countries: and epidemiological update based on data reported in 2014. Wkly Epidemiol Rec. 2016;22: 285–296. 27263128

[3] World Health Organization. Manual on case management and surveillance of the leishmaniases in the WHO European Region. 2017.

[4] Ready PD. Leishmaniasis emergence in Europe. Euro Surveill. 2010;15: 19505 20403308

[5] Arce A, Estirado A, Ordobas M, Sevilla S, García N, Moratilla L, et al Re-emergence of leishmaniasis in Spain: community outbreak in Madrid, Spain, 2009 to 2012. Euro Surveill. 2013;18: 20546 23929177 10.2807/1560-7917.es2013.18.30.20546

[6] Carrillo E, Moreno J, Cruz I. What is responsible for a large and unusual outbreak of leishmaniasis in Madrid? Trends Parasitol. 2013;29: 579–580. doi: 10.1016/j.pt.2013.10.007 24275160 10.1016/j.pt.2013.10.007

[7] Gradoni L. Epidemiological surveillance of leishmaniasis in the European Union: operational and research challenges. Euro Surveill. 2013;18: 20539 23929176 10.2807/1560-7917.es2013.18.30.20539

[8] Chappuis F, Rijal S, Soto A, Menten J, Boelaert M. A meta-analysis of the diagnostic performance of the direct agglutination test and rK39 dipstick for visceral leishmaniasis. BMJ. 2006;333: 723–0. doi: 10.1136/bmj.38917.503056.7C 16882683 10.1136/bmj.38917.503056.7C PMC1592383

[9] de Ruiter CM, van der Veer C, Leeflang MMG, Deborggraeve S, Lucas C, Adams ER. Molecular Tools for Diagnosis of Visceral Leishmaniasis: Systematic Review and Meta-Analysis of Diagnostic Test Accuracy. J Clin Microbiol. 2014;52: 3147–3155. doi: 10.1128/JCM.00372-14 24829226 10.1128/JCM.00372-14 PMC4313130

[10] Cota GF, de Sousa MR, Demarqui FN, Rabello A. The diagnostic accuracy of serologic and molecular methods for detecting visceral leishmaniasis in HIV infected patients: meta-analysis. PLoS Negl Trop Dis. 2012;6: e1665 doi: 10.1371/journal.pntd.0001665 22666514 10.1371/journal.pntd.0001665 PMC3362615

[11] Boelaert M, El-Safi S, Hailu A, Mukhtar M, Rijal S, Sundar S, et al Diagnostic tests for kala-azar: a multi-centre study of the freeze-dried DAT, rK39 strip test and KAtex in East Africa and the Indian subcontinent. Trans R Soc Trop Med Hyg. 2008;102: 32–40. doi: 10.1016/j.trstmh.2007.09.003 17942129 10.1016/j.trstmh.2007.09.003

[12] Boelaert M, Verdonck K, Menten J, Sunyoto T, van Griensven J, Chappuis F, et al Rapid tests for the diagnosis of visceral leishmaniasis in patients with suspected disease. Cochrane Database Syst Rev. 2014;6: CD009135. 10.1002/14651858.CD009135.pub2 PMC4468926 24947503

[13] Boelaert M, El Safi S, Mousa H, Githure J, Mbati P, Gurubacharya V l, et al Multi-centre evaluation of repeatability and reproducibility of the direct agglutination test for visceral leishmaniasis. Trop Med Int Health TM IH. 1999;4: 31–37. 10203171 10.1046/j.1365-3156.1999.00348.x

[14] Protocolos de la Red Nacional de Vigilancia Epidemiológica [Internet]. Red Nacional de Vigilancia Epidemiológica, Instituto de Salud Carlos III, CIBER Epidemiología y Salud Pública, Ministerio de Economía y Competitividad, Ministerio de Sanidad, Servicios Sociales e Igualdad.; 2015. Available: http://www.isciii.es/ISCIII/es/contenidos/fd-servicios-cientifico-tecnicos/fd-vigilancias-alertas/fd-procedimientos/PROTOCOLOS_RENAVE-ciber.pdf [last accessed on January 19th, 2018].

[15] Institute of Tropical Medicine, Antwerp, Belgium. http://www.itg.be/files/docs/TTP/brochures/PDT_BR_0008_E_1.2.pdf [last accessed on January 19th, 2018].

[16] Bray RS. Immunodiagnosis of leishmaniasis. Leishmaniasis. pp. 177–182.

[17] Cruz I, Cañavate C, Rubio JM, Morales MA, Chicharro C, Laguna F, et al A nested polymerase chain reaction (Ln-PCR) for diagnosing and monitoring *Leishmania infantum* infection in patients co-infected with human immunodeficiency virus. Trans R Soc Trop Med Hyg. 2002;96 Suppl 1: S185–189. 12055836 10.1016/s0035-9203(02)90074-x

[18] Cruz I, Chicharro C, Nieto J, Bailo B, Cañavate C, Figueras M-C, et al Comparison of new diagnostic tools for management of pediatric Mediterranean visceral leishmaniasis. J Clin Microbiol. 2006;44: 2343–2347. doi: 10.1128/JCM.02297-05 16825347 10.1128/JCM.02297-05 PMC1489479

[19] Nunes MS with contributions from T, Heuer C, Marshall J, Sanchez J, Thornton R, Reiczigel J, et al. epiR: Tools for the Analysis of Epidemiological Data [Internet]. 2017. Available: https://CRAN.R-project.org/package=epiR [last accessed on January 19th, 2018].

[20] R Core Team. R: A Language and Environment for Statistical Computing [Internet]. Vienna, Austria: R Foundation for Statistical Computing; 2016 Available: https://www.R-project.org/ [last accessed on January 19th, 2018].

[21] World Health Organization. Visceral leishmaniasis rapid diagnostic test performance [Internet]. 2011. Available: http://www.who.int/leishmaniasis/resources/9789241502238/en/ [last accessed on January 19th, 2018].

[22] Abass E, Kang C, Martinkovic F, Semião-Santos SJ, Sundar S, Walden P, et al Heterogeneity of *Leishmania donovani* parasites complicates diagnosis of visceral leishmaniasis: comparison of different serological tests in three endemic regions. PloS One. 2015;10: e0116408 doi: 10.1371/journal.pone.0116408 25734336 10.1371/journal.pone.0116408 PMC4348478

[23] Varani S, Ortalli M, Attard L, Vanino E, Gaibani P, Vocale C, et al Serological and molecular tools to diagnose visceral leishmaniasis: 2-years’ experience of a single center in Northern Italy. Afrin F, editor. PLOS ONE. 2017;12: e0183699 doi: 10.1371/journal.pone.0183699 28832646 10.1371/journal.pone.0183699 PMC5568375

[24] ter Horst R, Tefera T, Assefa G, Ebrahim AZ, Davidson RN, Ritmeijer K. Field evaluation of rK39 test and direct agglutination test for diagnosis of visceral leishmaniasis in a population with high prevalence of human immunodeficiency virus in Ethiopia. Am J Trop Med Hyg. 2009;80: 929–934. 19478251

[25] Chappuis F, Sundar S, Hailu A, Ghalib H, Rijal S, Peeling RW, et al Visceral leishmaniasis: what are the needs for diagnosis, treatment and control? Nat Rev Microbiol. 2007;5: 873–882. doi: 10.1038/nrmicro1748 17938629 10.1038/nrmicro1748

[26] Bhattacharyya T, Boelaert M, Miles MA. Comparison of Visceral Leishmaniasis Diagnostic Antigens in African and Asian *Leishmania donovani* Reveals Extensive Diversity and Region-specific Polymorphisms. Büscher P, editor. PLoS Negl Trop Dis. 2013;7: e2057 doi: 10.1371/journal.pntd.0002057 23469296 10.1371/journal.pntd.0002057 PMC3585016

[27] Kuhls K, Alam MZ, Cupolillo E, Ferreira GE, Mauricio IL, Oddone R, Feliciangeli MD, Wirth T, Miles MA, Schönian G. Comparative microsatellite typing of new world *Leishmania infantum* reveals low heterogeneity among populations and its recent old world origin. PLoS Negl Trop Dis. 2011 6;5(6):e1155 doi: 10.1371/journal.pntd.0001155 21666787 10.1371/journal.pntd.0001155 PMC3110170

[28] Gouzelou E, Haralambous C, Antoniou M, Christodoulou V, Martinković F, Živičnjak T, Smirlis D, Pratlong F, Dedet JP, Özbel Y, Toz SÖ, Presber W, Schönian G, Soteriadou K. Genetic diversity and structure in *Leishmania infantum* populations from southeastern Europe revealed by microsatellite analysis. Parasit Vectors. 2013 12 5;6:342 doi: 10.1186/1756-3305-6-342 24308691 10.1186/1756-3305-6-342 PMC4029556

[29] Kyriakou DS, Alexandrakis MG, Passam FH, Kourelis TV, Foundouli P, Matalliotakis E, et al Quick detection of *Leishmania* in peripheral blood by flow cytometry. Is prestorage leucodepletion necessary for leishmaniasis prevention in endemic areas? Transfus Med. 2003;13: 59–62. 12694549 10.1046/j.1365-3148.2003.00420.x

[30] Chitimia L, Muñoz-García CI, Sánchez-Velasco D, Lizana V, del Río L, Murcia L, et al Cryptic Leishmaniosis by *Leishmania infantum*, a feature of canines only? A study of natural infection in wild rabbits, humans and dogs in southeastern Spain. Vet Parasitol. 2011;181: 12–16. doi: 10.1016/j.vetpar.2011.04.016 21592669 10.1016/j.vetpar.2011.04.016

[31] Riera C, Fisa R, López-Chejade P, Serra T, Girona E, Jiménez M, et al Asymptomatic infection by *Leishmania infantum* in blood donors from the Balearic Islands (Spain). Transfusion (Paris). 2008;48: 1383–1389. 10.1111/j.1537-2995.2008.01708.x 18422844

[32] Horrillo L, San Martín JV, Molina L, Madroñal E, Matía B, Castro A, et al Atypical presentation in adults in the largest community outbreak of leishmaniasis in Europe (Fuenlabrada, Spain). Clin Microbiol Infect Off Publ Eur Soc Clin Microbiol Infect Dis. 2015;21: 269–273. 10.1016/j.cmi.2014.10.017 25658537

[33] Hailu A, Berhe N. The performance of direct agglutination tests (DAT) in the diagnosis of visceral leishmaniasis among Ethiopian patients with HIV co-infection. Ann Trop Med Parasitol. 2002 1;96(1):25–30. doi: 10.1179/000349802125000475 11989530 10.1179/000349802125000475

[34] Barbosa Júnior WL, Ramos de Araújo PS, Dias de Andrade L, Aguiar Dos Santos AM, Lopes da Silva MA, Dantas-Torres F, Medeiros Z. Rapid Tests and the Diagnosis of Visceral Leishmaniasis and Human Immunodeficiency Virus/Acquired Immunodeficiency Syndrome Coinfection. Am J Trop Med Hyg. 2015 11;93(5):967–9. doi: 10.4269/ajtmh.14-0798 26416105 10.4269/ajtmh.14-0798 PMC4703249

[35] Cota GF, de Sousa MR, de Freitas Nogueira BM, Gomes LI, Oliveira E, Assis TS, de Mendonça AL, Pinto BF, Saliba JW, Rabello A. Comparison of parasitological, serological, and molecular tests for visceral leishmaniasis in HIV-infected patients: a cross-sectional delayed-type study. Am J Trop Med Hyg. 2013 9;89(3):570–7. doi: 10.4269/ajtmh.13-0239 23836568 10.4269/ajtmh.13-0239 PMC3771302

[36] Basset D, Faraut F, Marty P, Dereure J, Rosenthal E, Mary C, Pratlong F, Lachaud L, Bastien P, Dedet JP. Visceral leishmaniasis in organ transplant recipients: 11 new cases and a review of the literature. Microbes Infect. 2005 10;7(13):1370–5. doi: 10.1016/j.micinf.2005.06.002 16046170 10.1016/j.micinf.2005.06.002

[37] Antinori S, Cascio A, Parravicini C, Bianchi R, Corbellino M. Leishmaniasis among organ transplant recipients. Lancet Infect Dis. 2008 3;8(3):191–9. doi: 10.1016/S1473-3099(08)70043-4 18291340 10.1016/S1473-3099(08)70043-4

[38] Campos-Varela I, Len O, Castells L, Tallada N, Ribera E, Dopazo C, Vargas V, Gavaldà J, Charco R. Visceral leishmaniasis among liver transplant recipients: an overview. Liver Transpl. 2008 12;14(12):1816–9. doi: 10.1002/lt.21538 19025932 10.1002/lt.21538

[39] Alonso S, Tachfouti N, Najdi A, Sicuri E, Picado A. Cost-effectiveness of diagnostic-therapeutic strategies for paediatric visceral leishmaniasis in Morocco. BMJ Glob Health. 2017 8 19;2(3):e000315 doi: 10.1136/bmjgh-2017-000315 29018581 10.1136/bmjgh-2017-000315 PMC5620433

[40] Kohanteb J, Ardehali S. Cross-Reaction of Sera from Patients with Various Infectious Diseases with *Leishmania infantum*. Med Princ Pract. 2008;14: 79–82. 10.1159/000083915 15785097

[41] Vilajeliu Balagué A, de Las Heras Prat P, Ortiz-Barreda G, Pinazo Delgado MJ, Gascón Brustenga J, Bardají Alonso A. [Imported parasitic diseases in the immigrant population in Spain]. Rev Esp Salud Publica. 2014;88: 783–802. doi: 10.4321/S1135-57272014000600010 25418568 10.4321/S1135-57272014000600010

[42] Bangert M, Molyneux DH, Lindsay SW, Fitzpatrick C, Engels D. The cross-cutting contribution of the end of neglected tropical diseases to the sustainable development goals. Infect Dis Poverty. 2017;6. 10.1186/s40249-017-0288-0 PMC5379574 28372566

