# Supplementary material for: Validation of rK39 immunochromatographic test and direct agglutination test for the diagnosis of Mediterranean visceral leishmaniasis in Spain
Source: PLoS Negl Trop Dis. 2018 Mar 1;12(3):e0006277. doi: 10.1371/journal.pntd.0006277 (PMC5849364; doi:10.1371/journal.pntd.0006277)
Supplement: S1 Fig — Details on the classification and workflow for testing samples in this study. (PPTX) [file pntd.0006277.s002.pptx]

## Slide 1
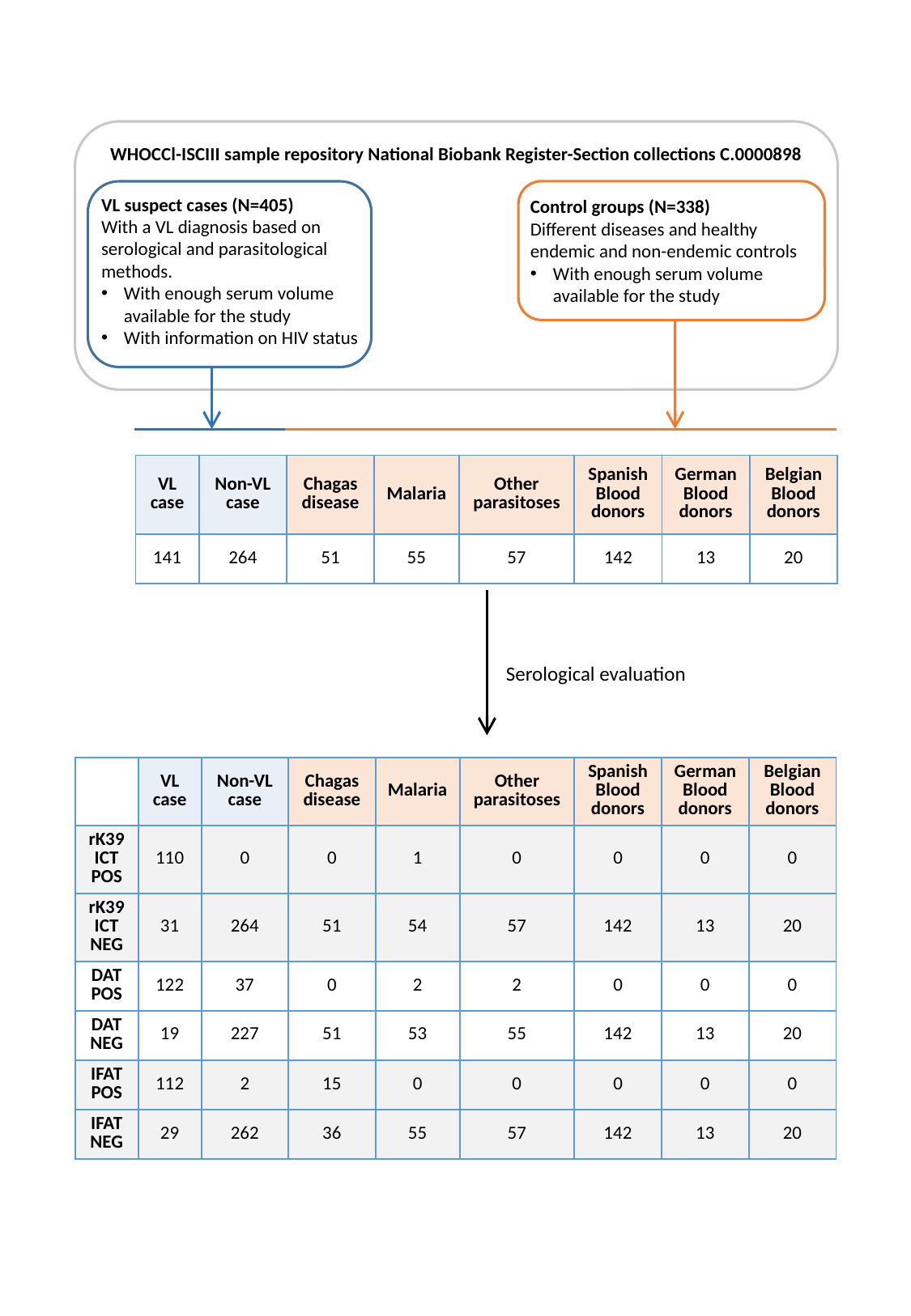

WHOCCl-ISCIII sample repository National Biobank Register-Section collections C.0000898
VL suspect cases (N=405)
With a VL diagnosis based on serological and parasitological methods.
With enough serum volume available for the study
With information on HIV status
Control groups (N=338)
Different diseases and healthy endemic and non-endemic controls
With enough serum volume available for the study
| VL case | Non-VL case | Chagas disease | Malaria | Other parasitoses | Spanish Blood donors | German Blood donors | Belgian Blood donors |
| --- | --- | --- | --- | --- | --- | --- | --- |
| 141 | 264 | 51 | 55 | 57 | 142 | 13 | 20 |
Serological evaluation
| | VL case | Non-VL case | Chagas disease | Malaria | Other parasitoses | Spanish Blood donors | German Blood donors | Belgian Blood donors |
| --- | --- | --- | --- | --- | --- | --- | --- | --- |
| rK39 ICT POS | 110 | 0 | 0 | 1 | 0 | 0 | 0 | 0 |
| rK39 ICT NEG | 31 | 264 | 51 | 54 | 57 | 142 | 13 | 20 |
| DAT POS | 122 | 37 | 0 | 2 | 2 | 0 | 0 | 0 |
| DATNEG | 19 | 227 | 51 | 53 | 55 | 142 | 13 | 20 |
| IFAT POS | 112 | 2 | 15 | 0 | 0 | 0 | 0 | 0 |
| IFAT NEG | 29 | 262 | 36 | 55 | 57 | 142 | 13 | 20 |
